# Metabolomics study of COVID-19 patients in four different clinical stages

**DOI:** 10.1038/s41598-022-05667-0

**Published:** 2022-01-31

**Authors:** Alberto Valdés, Lorena Ortega Moreno, Silvia Rojo Rello, Antonio Orduña, David Bernardo, Alejandro Cifuentes

**Affiliations:** 1 Laboratory of Foodomics, Institute of Food Science Research, CIAL, CSIC, Nicolás Cabrera 9, 28049 Madrid, Spain; 2 Dpt. Medicina, Universidad Autónoma de Madrid, Madrid, Spain; 3 Instituto de Investigación Sanitaria Hospital Universitario de La Princesa, Madrid, Spain; 4 Centro de Investigación Biomédica en Red (CIBERehd), Barcelona, Spain; 5 Servicio de Microbiología, Hospital Clínico Universitario de Valladolid, 47004 Valladolid, Spain; 6 Departamento de Microbiología, Universidad de Valladolid, Valladolid, Spain; 7 Unidad de Excelencia Instituto de Biomedicina y Genética Molecular (IBGM), Universidad de Valladolid-CSIC, Valladolid, Spain

**Keywords:** Viral infection, Mass spectrometry

## Abstract

SARS-CoV-2 (severe acute respiratory syndrome coronavirus 2) is the coronavirus strain causing the respiratory pandemic COVID-19 (coronavirus disease 2019). To understand the pathobiology of SARS-CoV-2 in humans it is necessary to unravel the metabolic changes that are produced in the individuals once the infection has taken place. The goal of this work is to provide new information about the altered biomolecule profile and with that the altered biological pathways of patients in different clinical situations due to SARS-CoV-2 infection. This is done via metabolomics using HPLC–QTOF–MS analysis of plasma samples at COVID-diagnose from a total of 145 adult patients, divided into different clinical stages based on their subsequent clinical outcome (25 negative controls (non-COVID); 28 positive patients with asymptomatic disease not requiring hospitalization; 27 positive patients with mild disease defined by a total time in hospital lower than 10 days; 36 positive patients with severe disease defined by a total time in hospital over 20 days and/or admission at the ICU; and 29 positive patients with fatal outcome or deceased). Moreover, follow up samples between 2 and 3 months after hospital discharge were also obtained from the hospitalized patients with mild prognosis. The final goal of this work is to provide biomarkers that can help to better understand how the COVID-19 illness evolves and to predict how a patient could progress based on the metabolites profile of plasma obtained at an early stage of the infection. In the present work, several metabolites were found as potential biomarkers to distinguish between the end-stage and the early-stage (or non-COVID) disease groups. These metabolites are mainly involved in the metabolism of carnitines, ketone bodies, fatty acids, lysophosphatidylcholines/phosphatidylcholines, tryptophan, bile acids and purines, but also omeprazole. In addition, the levels of several of these metabolites decreased to “normal” values at hospital discharge, suggesting some of them as early prognosis biomarkers in COVID-19 at diagnose.

## Introduction

SARS-COV-2 (severe acute respiratory syndrome coronavirus 2) is extremely infectious and has triggered a global pandemic. Infection of the lungs and human respiratory tract by this coronavirus leads to fever, myalgia and cough, and in some patients to acute respiratory distress syndrome (ARDS). While most patients experience very mild-to-moderate symptoms, around one in five patients develop pneumonia coupled with severe respiratory distress. These patients require treatment in hospital intensive care units (ICU), where infection can lead to multi-organ dysfunction, failure, and sometimes death. The COVID-19 (coronavirus disease 2019) pandemic has led to urgent and intense investigations of this disease, its causative agent, and its interaction with the human host. However, there are still many difficulties for an accurate SARS-CoV-2 patient’s risk categorization, which are consequences of COVID-19 complexity since coronavirus infection reflects a broad spectrum of patient symptoms, and as a result, diverse pathophysiological pathways are perturbed during the disease course. This complexity has taken to many groups to investigate this exciting topic using metabolomics, given that the circulating metabolome provides a snapshot of the physiological state of the organism^1,2^.

Although nuclear magnetic resonance (NMR) has been employed in a few metabolomics works^3^, mass spectrometry (MS)-based metabolomics has been the technique of choice to seek potential diagnostics biomarker candidates in COVID-19 disease. Although many of these works have been done using MS coupled to liquid chromatography (LC), the use of gas chromatography coupled to MS has also shown to provide interesting results about the illness evolution^4^. Many topics have been addressed regarding COVID-19 disease using metabolomics, for instance, metabolomics has displayed sex-specific metabolic shifts in non-severe COVID-19 patients during recovery process, showing that the major plasma metabolic changes were fatty acids in men and glycerophosphocholines and carbohydrates in women^5^. Metabolomics has also shown that it is possible to differentiate plasma metabolite profiles of COVID-19 survivors with abnormal pulmonary function from those of healthy donors or subjects with normal pulmonary function. These alterations mainly involved amino acid and glycerophospholipid metabolic pathways, increased levels of triacylglycerols (TG), phosphatidylcholines (PC), prostaglandin E2, arginine, and decreased levels of betain and adenosine^6^. Since many issues regarding the immune, both innate and adaptive, response remains unclear, they are subject to ongoing multi-omic investigations^1^, as well as comprehensive meta-analysis of global metabolomics datasets of COVID-19^2^. Metabolomics also showed that more than 100 lipids including glycerophospholipid, sphingolipids, and fatty acids (FA) were downregulated in COVID-19 patient sera, probably because of damage to the liver, which is also reflected in aberrancy in bilirubin and bile acids^7^. Significant differences were also determined between COVID-19 patients and healthy controls in terms of purine, glutamine, leukotriene D4 (LTD4), and glutathione metabolisms. Decrease levels were determined in R‐S lactoglutathione and glutamine, and increase levels were detected for hypoxanthine, inosine, and LTD4^8^).

As mentioned above, there are still many difficulties for an adequate categorization of SARS-COV-2 patients through the use of potential metabolic markers of clinical severity identified at the beginning of the COVID-19 disease, reason why many different works have addressed this challenging topic. Thus, using high-throughput omics, the dynamic changes in the metabolome (and proteome) profile of non/severe to severe disease cohorts were studied, and they could be used to predict the disease development: for example, the simultaneous decline in the levels of malic acid and glycerol 3-phosphate in healthy to mild to fatal groups^9^. On the other hand, the level of guanosine monophosphate was found to be modulated along with carbamoyl phosphate in mild to severe patients, suggesting the role of immune dysfunction and nucleotide metabolism in the progression of non/severe COVID-19 to severe condition^9^.

Danlos et al. also reported alterations in the plasma metabolome reflecting the clinical presentation of COVID-19 patients with mild (ambulatory) diseases, moderate disease (radiologically confirmed pneumonitis, hospitalization and oxygen therapy), and critical disease (in intensive care)^10^; and altered tryptophan metabolism into the kynurenine pathway has been related to inflammation and immunity in critical COVID-19 patients in comparison to mild disease patients^10^. Increased levels of kynurenine and decreased levels of arginine, sarcosine and LPC were also observed as the top-performing metabolites for identifying COVID-19 positive patients from healthy control subjects^11^. The role of the tryptophan-nicotinamide pathway, linked to inflammatory signals and microbiota, and the involvement of cytosine were also described as possible markers to discriminate and predict the disease evolution^12^.

Xiao et al. characterized the globally dysregulated metabolic pathways and cytokine/chemokine levels in COVID-19 patients compared to healthy controls. They identified the escalated correlations between circulating metabolites and cytokines/chemokines from mild to severe patients, and revealed the disturbed metabolic pathways linked to hyper-inflammation in severe COVID-19, demonstrating that arginine, tryptophan, or purine metabolism modulates the inflammatory cytokine release^13^.

As can be deduced from above and other works^14–16^, the biological mechanisms involved in SARS-CoV-2 infection are only partially understood. Thus in the current work we have explored the plasma metabolome of non-COVID controls as well as 145 COVID patients at diagnose through reverse phase liquid chromatography coupled to quadrupole-time of flight mass spectrometry (RP/HPLC-qTOF MS/MS) analysis. Moreover, patients were stratified based on their clinical evolution in asymptomatic (not requiring hospitalization), patients with mild disease (defined by a total time in hospital lower than 10 days), patients with severe disease (defined by a total time in hospital over 20 days and/or admission at the ICU) and patients with fatal outcome or deceased. In addition, follow up samples between 2 and 3 months after hospital discharge were also obtained from the hospitalized patients with mild prognosis to investigate the disease sequels in the metabolome and how the recovery is reflected in the altered biological pathways. The final goal of the currents work is to find biomarkers that will increase our understanding about how the COVID-19 illness evolves and will improve our prediction about how a patient could progress based on the metabolites profile of plasma obtained at an early stage of the infection.

## Results

### Metabolite identification

To yield a wider view of the metabolomics changes during the course of disease in COVID-19 patients, an untargeted metabolomics analysis based on RP/HPLC-qTOF MS/MS analysis using two different ionization modes (ESI (+) and ESI (−)) was applied to increase the coverage of identified metabolites. Data obtained from each ESI ionization mode were processed independently to avoid intensity-bias. After data post-processing, the RP/HPLC-qTOF MS/MS analysis resulted in the annotation of 203 metabolites: 117 in ESI (+), 70 in ESI (−) and 16 in both ionization modes. The full list of identified metabolites for each ionization mode together with the statistical values for the different analyses (ANOVA, U test and PLS-DA) is shown in Supplementary Tables S1 and S2.

### Analysis of samples at hospital admission

**Figure 1:**
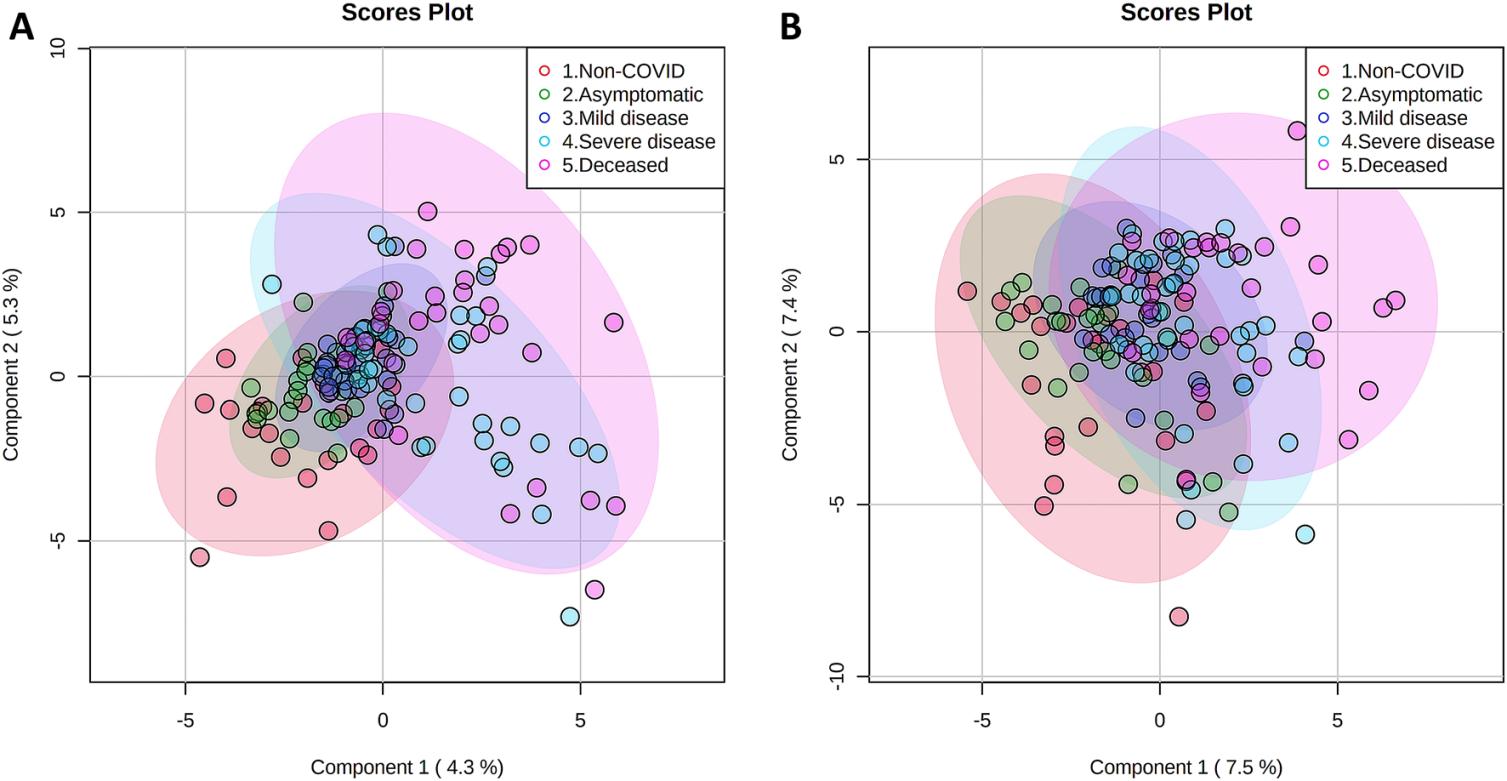
PLS-DA score plots of data obtained by RP/HPLC-qTOF MS/MS ESI (+) (**A**) and RP/HPLC-qTOF MS/MS ESI (–) (**B**) from plasma of patients collected at hospital admission.

The PCA analysis from both ESI (+) and ESI (−) ionization modes of samples collected at hospital admission showed no differences between the non-COVID control and/or the different COVID-19 positive groups (Supplementary Fig. S1A,B). On the other hand, and even though that the “Leave-one-out” cross-validation method indicated that the percentage of variation explained and the predictive ability of the models was rather low (R^2^Y = 0.591 and Q^2^ = 0.327 for ESI (+); R^2^Y = 0.540 and Q^2^ = 0.325 for ESI (−)), the PLS-DA analysis suggested that asymptomatic and mild disease groups were closer to the non-COVID control group; and patients with severe disease were closer to the deceased group (Fig. 1A,B). This analysis also showed 17 and 11 metabolites with VIP score > 1.5 in ESI (+) and ESI (−) modes, respectively (Supplementary Tables S1 and S2). It is important to note that the abundance of most of these compounds increased with the COVID-19 severity stage (Supplementary Fig. S2).

The Kruskal–Wallis ANOVA test of ESI (+) data showed 35 metabolites which abundance was significantly altered, whereas 30 metabolites were altered in ESI (−) mode (Supplementary Tables S1 and S2). The heatmap representation of these altered metabolites showed similar results as those obtained in the PLS-DA analysis: the asymptomatic and mild disease COVID-19 patients were closer to the non-COVID control group; and the severe disease patients were closer to the deceased group (Supplementary Fig. S3A,B). Most of the significantly altered metabolites after the ANOVA analysis already presented a VIP score > 1.5 in the PLS-DA analysis, and a few more were obtained.

The correlation analysis showed different sets of metabolites with similar abundancy among the analyzed groups. In ESI (+), several acylcarnitines (3-hydroxybutyrylcarnitine, hexanoyl-l-carnitine, decanoyl-l-carnitine, octanoyl-l-carnitine, arachidonoyl-l-carnitine, linoleoylcarnitine, acetyl-l-carnitine, lauroylcarnitine, oleoyl-l-carnitine and palmitoyl-l-carnitine), PC/LPC compounds (2-lysophosphatidylcholine, PC (p-16:0/0:0), LPC (o-16:0), LPC (20:2), PC (18:1/16:0), LPC (16:0), LPC (p-18:0), LPC (17:0), PC (18:2e) PC (18:1e) and PC (20:4e)), and amino acids (tryptophan, L-valine, L-isoleucine, L-methionine and L-tyrosine), were grouped together; whereas in ESI (−), the most relevant sets were composed by LPC, FA derivatives or bile acids (glycodeoxycholic acid, taurodeoxycholic acid, glycocholic acid, glycoursodeoxycholic acid and taurocholic acid). The Pearson correlation (r) values and the respective p-values are presented in Supplementary Tables S3 and S4 for ESI (+), and Supplementary Tables S5 and S6 for ESI (−).

The Mann–Whitney U test between the different COVID-19 positive groups and the non-COVID control group showed 8 metabolites altered in the asymptomatic group (5 of them with increased values and 3 with decreased values). The number of significantly altered metabolites increased to 26 in the mild disease group, 14 and 12 with increased and decreased values, respectively. Some of them were already observed as altered in the asymptomatic group, such as S-methyl-3-thioacetaminophen and nicotinamide riboside cation, which values increased more in the mild disease group; and N-methyl-2-pyrrolidone, trimethoprim and L-methionine, which values continued to decrease in the mild disease group. In the severe disease group, the number of significantly altered metabolites rose to 45 (32 with increased and 13 with decreased values); and for the deceased group, the total number of altered metabolites was 35 (23 with increased values and 12 with decreased values). Many of these metabolites were already observed as significant in the previous PLS-DA and ANOVA analyses.

**Figure 2:**
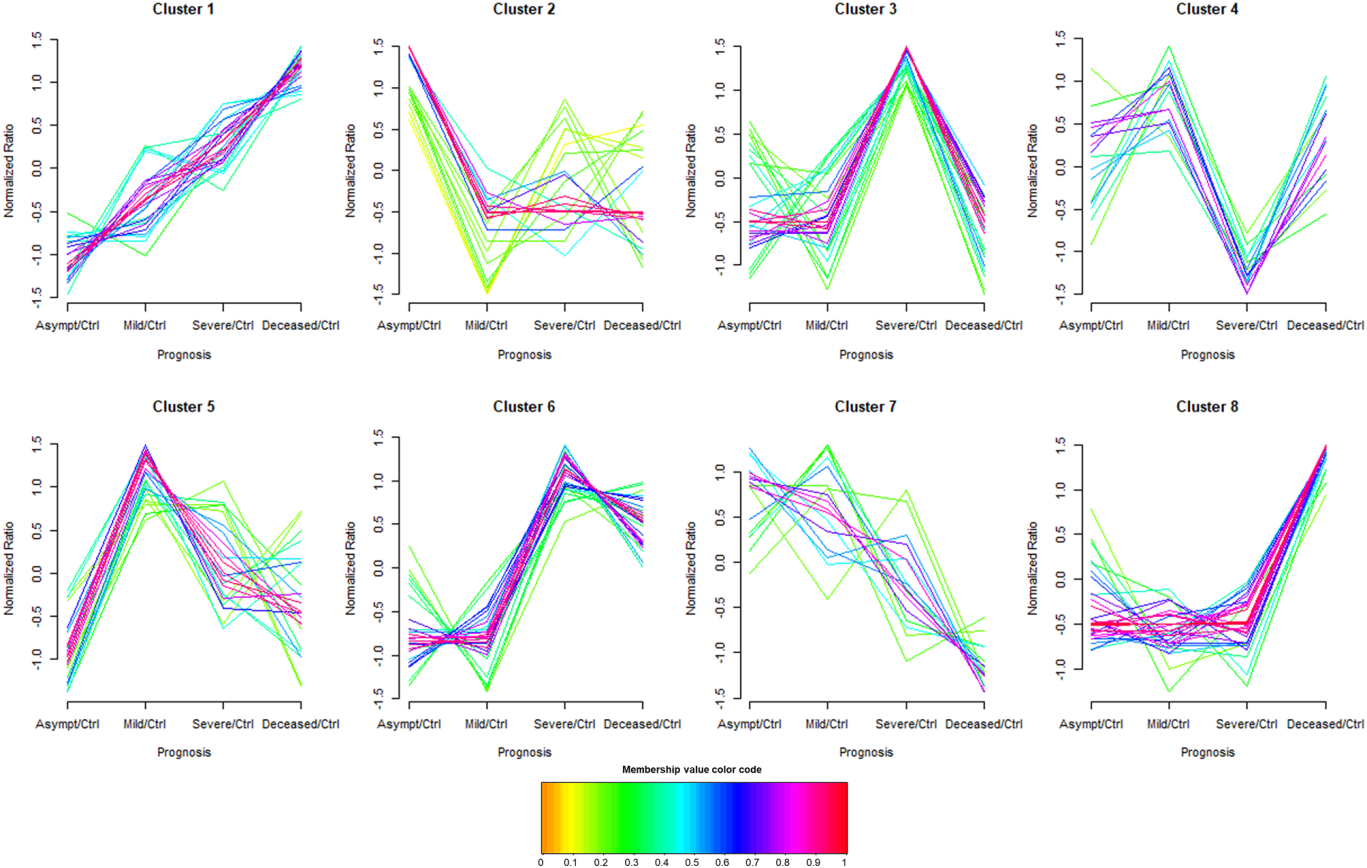
Fuzzy c-means clustering patterns of metabolite ratios between COVID-19 positive patients (asymptomatic, mild, severe and deceased) and non-COVID-19 control patients (Ctrl). Each trace is colour coded according to its membership value for the respective cluster (see colour bar).

The further analysis of the fold change ratio patterns obtained in the previous comparisons suggested 8 main clusters to be formed (Fig. 2, the MFuzz membership value and cluster composition are shown in Supplementary Tables S7 and S8).

This analysis provides is a general overview of groups of metabolites with similar alteration patterns between the different groups of samples (after normalization by the non-COVID control group) and allows the combination of ESI (+) and ESI (−) data together since the normalization using the non-COVID control group eliminates the bias derived from the use of different ESI ionization modes. It has to be noted that this analysis does not take into account the statistical differences after a non-parametric Mann–Whitney U test between the COVID-19 positive samples and the control group, as it only clusters the metabolites according to their fold change ratio similarity. Among the identified clusters, cluster 1 represented those metabolites which abundance continuously increased from the asymptomatic to the deceased group, and included 3-hydroxybutyrylcarnitine, glycocholic acid, LPE (22:6), nervonic acid and palmitic acid, among others. On the other hand, cluster 7 represented those metabolites which abundance continuously decreased from the asymptomatic to the deceased group, and was composed by three PC (PC (16:0/20:4), PC (20:4e) and PC (20:5e)) and tryptophan. Based on the previous PLS-DA and ANOVA results, metabolites of cluster 6 were of special interest because their abundance was highest in the severe disease and the deceased groups. This cluster included 2-lysophosphatidylcholine, alpha-linolenic acid, linoleic acid or L-isoleucine, methyl ester. Finally, cluster 8 was the most crowded (24 metabolites) and was composed by metabolites which abundance mainly increased in the deceased group. Some of these metabolites were adipoyl-l-carnitine, glycodeoxycholic acid and taurodeoxycholic acid.

In order to provide the chemical classes significantly altered in the different group comparisons, a chemical enrichment analysis using ChemRICH was performed. No chemical classes were significantly altered in the comparison between the asymptomatic and the non-COVID control group; but the chemical class “carnitines” was increased, and the “unsaturated lysophosphatidylcholines” was altered (some species increased, others decreased) in the mild disease group. In the case of the severe disease group, “androstenols” were significantly decreased (considering 3 metabolites); xanthines were significantly altered with some species increased, others decreased; and 2-pyridinylmethylsulfinylbenzimidazoles, pyridines and unsaturated fatty acids were significantly increased. This last chemical class was also increased in the deceased group, based on the abundance of nervonic acid, linoleic acid, alpha-linolenic acid, trans-vaccenic acid and palmitoleic acid. The whole list of chemical classes and the respective p-values obtained for each comparison is presented in Supplementary Tables S9–S11, and the representations of all the annotated metabolites onto biochemical networks, constructed using chemical and biochemical similarities from MetaMapp, is shown in Supplementary Figs. S4–S7.

Finally, metabolite set enrichment analysis using the significantly altered metabolites in each comparison was performed using MBROLE 2.0 (Table 1). It has to be noted that only 5, 17, 28 and 16 altered metabolites in the asymptomatic, mild disease, severe disease and deceased groups, respectively, could be mapped with valid KEGG IDs (many carnitines and omeprazole derivatives could not be mapped).

**Table 1:** Significantly enriched KEGG human metabolic pathways (in dark shade) from the analysis of significantly altered metabolites (after Mann–Whitney U test with p-value < 0.05) in asymptomatic, mild disease, severe disease and deceased COVID-19 positive groups as compared to non-COVID control patients.

Pathway name	Asymptomatic/non-COVID		Mild disease/non-COVID		Severe disease/non-COVID		Deceased/non-COVID	
	P-value	Metabolites	P-value	Metabolites	P-value	Metabolites	P-value	Metabolites
Phenylalanine metabolism	0.0566	Salicylic acid	0.2197	Phenylacetyl-l-glutamine	0.0488	Hippuric acid 3-Hydroxyphenylacetate	0.0236	Hippuric acid 3-Hydroxyphenylacetic acid
Epithelial cell signaling in Helicobacter pylori infection			0.0265	Urea	0.038	Urea	0.0258	Urea
Synthesis and degradation of ketone bodies			0.0317	3-Hydroxybutyric acid			0.0308	3-Hydroxybutyric acid
Biosynthesis of unsaturated fatty acids					0.0082	Linoleic acid alpha-Linolenic acid Nervonic acid	0.0317	Alpha-linolenic acid Nervonic acid
Purine metabolism			0.0128	Urea Inosine Xanthine	0.0337	Urea Inosine Xanthine	0.0822	Urea Xanthine
Caffeine metabolism			0.1069	Xanthine	0.0005	Xanthine Theophylline Caffeine	0.1039	Xanthine
Alpha-Linolenic acid metabolism			0.194	PC (18:1/16:1)	0.0378	PC (18:1/16:1) alpha-Linolenic acid	0.1888	alpha-linolenic acid
Linoleic acid metabolism			0.1307	PC (18:1/16:1)	0.0169	PC (18:1/16:1) Linoleic acid		

This analysis showed that the *Phenylalanine metabolism* and the *Biosynthesis of unsaturated fatty acids* were enriched in the severe disease and deceased groups. In the case of *Phenylalanine metabolism,* 3-hydroxyphenylacetic acid had increased levels and hippuric acid had decreased levels in both comparisons. In the case of *Biosynthesis of unsaturated fatty acids*, alpha-linolenic acid, nervonic acid and linoleic acid had increased values in both groups. Another enriched pathway in the mild and severe disease groups was the *Purine metabolism*. Three metabolites were found altered that matched this pathway (urea, inosine and xanthine), all of them with increased values in both COVID-19 positive groups. Finally, the *Caffeine metabolism* pathway was enriched in the severe disease group, based on the abundance of xanthine and caffeine (with increased values) and theophylline (with decreased values).

### Follow up study of mild disease patients at hospital discharge

The second statistical approach consisted on the comparison of plasma metabolites from patients belonging to the mild disease group: at hospital admission and after 2–3 months of hospital discharge. The PCA and PLS-DA analyses could separate between the two groups of samples (Supplementary Figs. S8 and S9), but the PLS-DA analysis was more informative (Supplementary Tables S12 and S13). In ESI (+) data, 13 compounds had VIP scores > 1.5, 6 of them with increased values (kelevan, LPC (14:0), phenylacetyl-l-glutamine, bilirubin, L-methionine and LPC (16:1)), and 7 with decreased values (hypoxanthine, inosine, LPC (p-18:0), acetaminophen sulfate, myclobutanil, 1-methyladenosine and L-tryptophanamide). In the case of ESI (−), 11 compounds had a VIP score > 1.5, 2 of them with increased values (methylsuccinic acid and LPC 18:2) and 9 with lower values (hypoxanthine, xanthine, 3-hydroxybutyric acid, 3-hydroxybenzaldehyde, S-methyl-3-thioacetaminophen, acetaminophen sulfate, octadecanedioic acid, nervonic acid and LPE (20:4)). The graphical representation of these metabolites, together with the values of the control samples, indicated that the intensity of some of these metabolites got back to “normal” values at hospital discharge (Supplementary Fig. S10). However, it has to be noticed that these patients may have unique metabolic signatures that could be related to good or poor quality of life. In this regard, the medical history indicates that none of the patients had any symptoms of bad quality of life when the samples were taken after hospital discharge, and no sequels of the disease have been reported since then.

Moreover, the paired non-parametric U test showed 43 metabolites which abundance was significantly altered (19 of them with increased values and 24 with decreased values) in ESI (+); and in ESI (−), 35 metabolites were altered (13 and 22 were more and less abundant, respectively) at hospital discharge, confirming that all the metabolites with VIP scores > 1.5 after PLS-DA analysis were statistically significant between the two groups.

**Figure 3:**
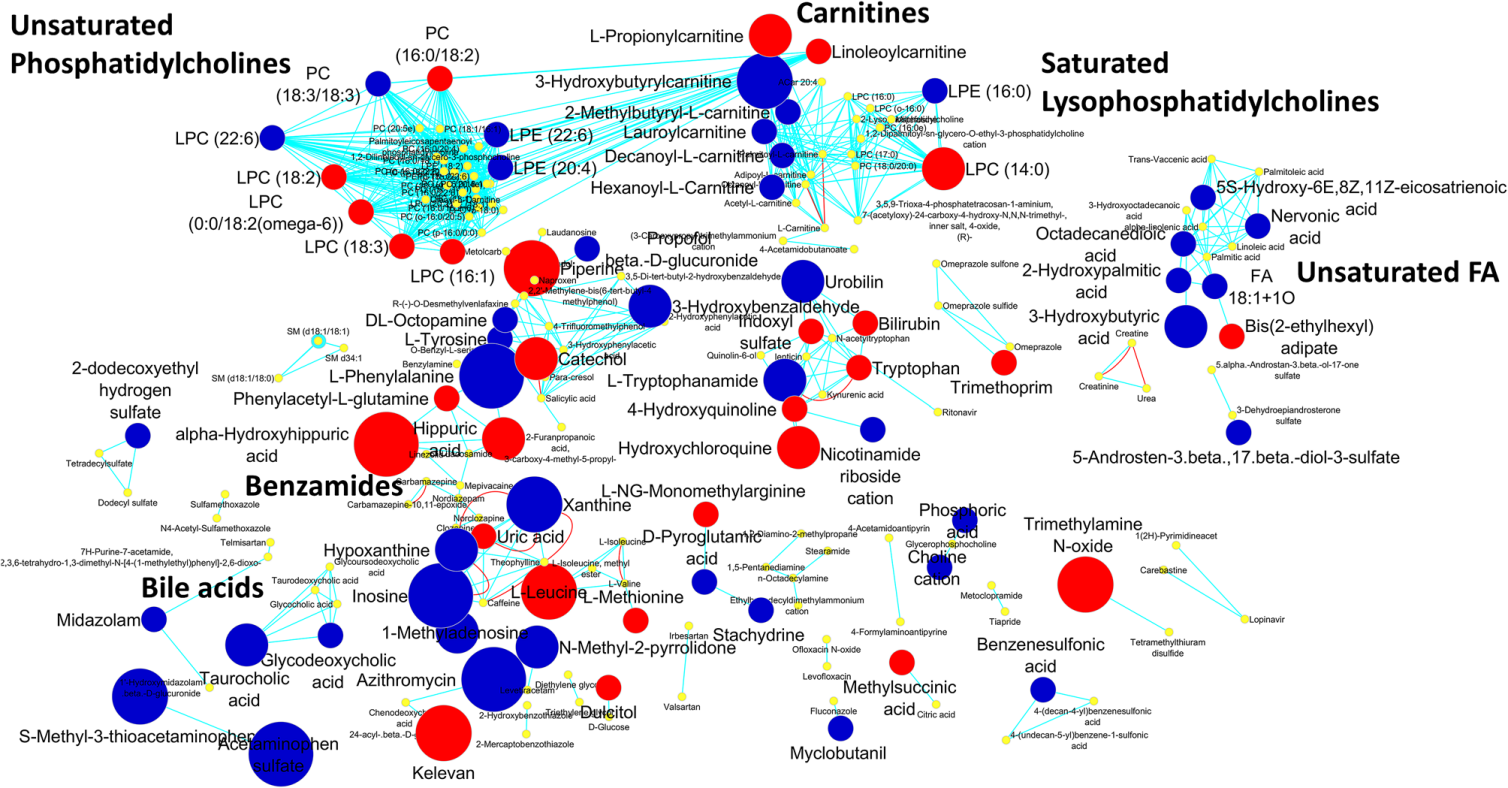
MetaMapp visualization of metabolomic data highlighting the differential metabolic regulation in mild disease COVID-19 positive patients at hospital discharge compared to the same patients at hospital admission. Red edges denote KEGG reactant pair links and light blue edges symbolize Tanimoto chemical similarity at T > 700. Node sizes reflect fold change. Metabolites found significantly increased are given as red nodes, and blue nodes denotes decreased metabolites (significance determined using Mann–Whitney U test with p-value < 0.05). Metabolites not significantly altered are given as yellow nodes.

The chemical class analysis and the biochemical overrepresentations of the metabolomics changes indicated that aromatic amino acids, carnitines, saturated laurates, and pyrrolidines were significantly decreased, while benzamides were significantly increased (Fig. 3 and Supplementary Table S14). Other chemical classes, such as unsaturated LPC were highly represented (7 metabolites), with some species increased and others decreased.

The metabolite set enrichment analysis of the significantly altered metabolites showed again that the *Phenylalanine metabolism* and the *Purine metabolism* (among others) were enriched in the mild disease group at hospital discharge as compared to the levels at hospital admission (Supplementary Table S15). It is interesting to note that all the considered metabolites (phenylalanine, hippuric acid, phenylacety-l-glutamine for the *Phenylalanine metabolism*; hypoxanthine, inosine, uric acid and xanthine for the *Purine metabolism*) had the opposite direction as when the mild disease group at hospital admission was compared to the non-COVID control group.

## Discussion

Since the COVID-19 pandemic took place in 2019, several studies have been published with the aim of investigating the metabolomics changes in serum from COVID-19 positive patients^1,2^. In most of those manuscripts, metabolomics and/or lipidomics approaches have been applied to different cohorts of patients, but only a few have included four different COVID-19 positive groups based on the total time of hospitalization (asymptomatic, less than 10 days, more than 20 days, and deceased), combined with a follow up study of COVID-19 mild disease patients at hospital discharge.

In the present work, and after the different statistical analyses, several metabolites could distinguish between the end-stage and the early-stage (or non-COVID) disease groups. These metabolites were mainly involved in the metabolism of carnitines, ketone bodies, fatty acids, LPC/PC, tryptophan, bile acids, purines and omeprazole. In addition, the levels of several of these metabolites decreased to “normal” values at hospital discharge, suggesting some of them as early prognosis biomarkers in COVID-19 at diagnose. The alteration of some of these metabolites have been already observed in previous studies, but there still exist some discrepancies between them.

One group of metabolites observed with a good Pearson correlation among the different analysed samples are acylcarnitines. Acylcarnitines are involved in the entrance of FAs to the mitochondria for β-oxidation and energy production. These metabolites are translocated across the inner mitochondrial membrane and the carnitine palmitoyl transferase 2 removes carnitine from acylcarnitines regenerating acyl-coAs. In the liver, acyl-CoA participates in β-oxidation with the final production of acetyl-CoA, and carnitine returns to the cytoplasm for another cycle^17^. Our results indicate that some of these metabolites were significantly increased in COVID-19 positive patients (such as carnitines with long-chain fatty acyl groups in the mild disease group, and medium-chain acylcarnitines in the deceased group). These results are in good agreement with previous results where the levels of acylcarnitines were increased^18^, but they are also contradictory to others, where acylcarnitines were decreased in COVID-19 patients^19^. Another study has reported that respiratory viruses, such as the influenza virus, promotes the accumulation of acylcarnitines^20^, and this might be the case for SARS-CoV-2, as we observed that the levels of most acylcarnitines decreased to “normal” values at hospital discharge. Moreover, during the β-oxidation process, ketone bodies such as 3-hydroxybutyric acid are produced. Ketone bodies provide an alternative fuel during fasting, post-exercise or pregnancy, but they also play roles as modulators of inflammation, immune cells function and oxidative stress^21^. We observed this metabolite as significantly increased in mild disease (p-val = 0.021) and deceased (p-val = 0.001) groups, and almost significantly increased in the severe disease group (p-val = 0.057). It has been reported that COVID-19 infection caused ketosis that increases the days of hospitalization and mortality^22^, and that abnormally levels of acetoacetic acid, 3-hydroxybutyric acid, and acetone are found in COVID-19 patients^23^. On the other hand, 3-hydroxybutyric acid, together with nicotinic acid, has shown to confer anti-inflammatory effects in TNF-α by decreasing the level of pro-inflammatory proteins (iNOS, COX-2) or secreted cytokines (IL-6 and IL-1β)^24^, which might explain the continuously increased levels of 3-hydroxybutyric acid observed in the different COVID-19 positive groups.

Our results also demonstrate that the levels of several UFAs (nervonic acid, linoleic acid, alpha-linolenic acid, trans-vaccenic acid and palmitoleic acid) correlated with worse prognosis of COVID-19; and the levels of these metabolites decreased in mild disease patients at hospital discharge. Among these FAs, linoleic acid has been involved in the inactivation of enveloped viruses, such as influenza; and the exogenous supplementation of it has demonstrated to suppress replication of the Middle East Respiratory Syndrome coronavirus (MERS-CoV)^25^. Even more, other studies focused on HCoV-229E coronavirus have also demonstrated that the host lipid metabolic remodelling, specially linoleic acid, was associated with the coronavirus propagation^26^. Specifically in SARS-CoV-2, linoleic acid can reduce the interaction of the virus spike protein with the ACE2 receptor^27^. In fact, it might stabilize the spike protein in a closed conformation blocking its interaction with ACE2^28^. Other polyunsaturated fatty acids (PUFAs) can also modify host membrane fluidity and inactivate viruses by disrupting their envelopes^29^. The changes in the membrane fluidity may distress the conformation of host and viral proteins and be determining for the SARS-CoV-2^27^. The mechanisms of how PUFAs inhibit the virus entry might be explained by the inhibition of endosomal proteases of the host. FAs adopt a flat conformation or spherical liposomal interface that could interrupt the contact between the host membrane and the viral envelope inhibiting SARS-CoV-2 attachment^27^. Therefore, in our results, a higher level of unsaturated fatty acids during the coronavirus infection might be explained by the PUFAs’ inhibitory effect on viral binding.

Another set of metabolites with a good Pearson correlation between analysed samples were LPC/PC compounds. Among these compounds, LPC (14:0 and 0:0/16:2(omega-6)) had a VIP score > 1.5 and their abundancy decreased with the worst prognosis of the infection; LPC (16:1) was significantly decreased in the mild disease group; LPC (18:2) was decreased in the mild disease and the deceased groups; and LPC (22:6) was decreased in the severe disease group. On the other hand, we observed that the levels of different LPEs (16:0 and 22:6) were increased in the end-stage disease groups. Previous studies have reported that separation of SARS-CoV-2 disease from healthy patients is marked by changes in lysophospholipids (LPs) and glycerophospholipids^30^, and the diminishment of several LPC (16:0, 18:0, 18:1 and 18:2) have been identified in COVID-19 positive patients^31^. The decrease of these molecules has been related to ARDS and sepsis in severely ill patients. However, other studies have shown an increase in the levels of LPC and LPE^18^. The alteration in lipid homeostasis of host cells is considered as a virus´ strategy for creating a good environment for replication. In this sense, PLA2 (a group of enzymes that hydrolyze phospholipids to yield FA and LPs) has been suggested as involved in coronavirus replication for the production of lysophospholipids that are required to form the membrane structure for the viral RNA synthesis^32^.

Previous studies have also demonstrated that tryptophan metabolism is altered in patients with COVID-19^10,15,33^. One important metabolite of the tryptophan metabolism is kynurenic acid. This metabolite was dramatically increased in patients in the end-stages of the infection, while tryptophan was significantly decreased in the same group of patients. Results from Danlos et al. (2021) have showed a decreased in tryptophan in COVID-19 severity stages, while its immunosuppressive metabolite kynurenine was increased in critical patients^10^. The decrease of tryptophan in critical patients compared to controls suggests a consume of tryptophan 2,3-dioxygenase and indoleamine 2,3-dioxygenase that produce the kynurenic acid precursor kynurenine. Other studies have showed that the increase of kynurenine is involved in inflammation and organ injury in SARS-CoV-2^33,34^. In addition, the increased ratio between kynurenine and trypthophan has been positively correlated with pro-inflammatory cytokines and poor prognosis of the COVID-19 infection^11,34–36^.

Another group of metabolites found altered in our study are related to the bile acid metabolism (taurocholic acid, taurodeoxycholic acid, glycodeoxycholic acid, glycocholic, and glycoursodeoxycholic acid). The levels of these metabolites mainly increased with the severity of the disease, and some of them got back to “normal” values at hospital discharge in the mild disease group. Increase levels of bile acids have been found in previous studies^7,15^. The main function of bile acids is to eliminate cholesterol and to facilitate the absorption of fat-soluble nutrients, but they can also act as signalling molecules to promote or inhibit virus replication^37^. It has been demonstrated that bile acids can limit in vitro replication of herpes simplex virus^38^ or influenza A virus^39^, or that they can promote in vitro replication of hepatitis B and C viruses^40^. Our results suggest that the last option might be the case for SARS-CoV-2, but it remains unclear the specific effects and molecular mechanisms.

Other metabolites that might be involved in SARS-CoV-2 replication are related with the purine metabolism, and it has been suggested that SARS-CoV-2 remodels host folate and one-carbon metabolism to support de novo purine synthesis^41^. However, purine metabolites have been also related with the inflammation process induced by SARS-CoV-2^13,15^. In the last study, the levels of xanthines correlated with pro-inflammatory cytokines, such as IL-6, in severe patients corroborating the link between the disturbances in metabolic pathways and hyper-inflammation in COVID-19. Our results demonstrate that different metabolites involved in the purine metabolism (urea and xanthine) increased with the severity stage, but others do not follow a clear trend (inosine). In any case, most of them got back to “normal” values at hospital discharge.

Apart from the endogenous metabolites, several xenobiotics and xenobiotic derivatives were observed as altered between the different groups of samples, but most of them were discarded because the clinical history of the patients was not considered when the sample cohort was selected. However, it is interesting to note that the levels of omeprazole and its derivatives were higher in patients with severe infection, but also in those asymptomatic patients infected by COVID-19, pointing to this xenobiotic as a possible marker in SARS-CoV-2 infection. This is of interest given that, although proton pump inhibitors do not make patients more susceptible to SARS-CoV-2 infections, recent observational studies have suggested that patients taking them may have an increased risk for severe COVID-19^42^, as we have here confirmed.

## Conclusion

In conclusion, COVID-19 positive patients display an alteration in the metabolism of carnitines, ketone bodies, fatty acids, LPC/PC, tryptophan, bile acids, purines and omeprazole, suggesting metabolites as 3-hydroxibutirate, linoleic acid, LPC (14:0 and 18:2), LPE (22:6), kynurenic acid and tryptophan as potential biomarkers of clinical severity. This work also shows that metabolomics is a highly valuable resource for a better understanding of the host metabolic responses associated with COVID-19, to expand our knowledge about the pathogenesis of patients under different symptomatic conditions, and to assist in the identification of disease biomarkers and the development of diagnostic assays, as well as possible therapeutic strategies^9^. The metabolomic strategies used to combat the pandemic could lay the foundation for a long-term plan for future outbreaks. Moreover, novel breakthroughs achieved through this omics (and multi-omics) approaches will not only aid to combat this pandemic but also propel wider adoption of these technologies by the scientific community and governmental institutions. Nevertheless, many questions regarding COVID-19 remain to be answered.

## Methods

### Patient recruitment and sample obtention

A total of 145 adult patients, over 18 years, who attended the A&E unit at “Hospital Clínico Universitario de Valladolid” (Valladolid, Spain) during the COVID-19 outbreak between March and April 2020 were recruited following ethics approval by the local ethics committee (Comité ético de investigación con medicamento -CEIm- de Valladolid este; PI20-1716). All research was performed in accordance with relevant guidelines/regulations. Informed consent was obtained from all participants. Samples from all patients were taken at hospital admission (without any standard of care) and, based on the medical history, none of the patients were treated with any drug for COVID-19-related symptoms.

Patients included 25 negative controls (non-COVID) who attended the hospital for non-COVID related issues (52% males, mean age 66.4 ± 9.6 years) and 120 COVID-19 patients as confirmed by a positive result for severe acute respiratory syndrome coronavirus 2 (SARS-CoV-2) infection by polymerase chain reaction on a nasopharyngeal sample. Of them, 28 were asymptomatic and did not require hospital admission (50% male, 65.2 ± 15.2 years); 27 had mild disease defined by a total time in hospital lower than 10 days (44% male, 65.3 ± 11.5 years); 36 had severe disease, defined by a total time in hospital over 25 days and/or admission at the ICU (66.7% male, 65.3 ± 11.5 years); and 29 patients with fatal outcome or deceased (45% male, 71.6 ± 8.4 years). Last, follow up samples between 2 and 3 months after hospital discharge were also obtained from the hospitalized patients with mild prognosis. Detailed demographics, co-morbidities and treatments of these patients can be found in Supplementary Table S16.

### Chemicals and reagents

LC–MS-grade acetonitrile (ACN) and methanol were obtained from VWR Chemicals (Barcelona, Spain), whereas ultrapure water was obtained from a Millipore system (Billerica, MA, USA). Formic acid was purchased from Fisher Scientific (Waltham, MA, USA).

In all cases, a blood sample (in heparin vacutainer blood tubes) was immediately acquired when the patients arrived at the A&E. Blood was preserved at 4 °C and processed within 24 h by performing a density gradient centrifugation over ficoll. Plasma was collected, aliquoted and preserved at − 80 °C until used.

### Metabolite extraction

Plasma samples from all patients were thaw on ice and vortex for 30 s. Thereafter, 100 µL of each sample was taken, 400 µL of methanol at -20 °C was added, and the mixture was vortexed for 1 min and incubated on ice for 10 min. Samples were then centrifuged at 14, 8000 rpm for 20 min at 4 °C, and 300 µL of the supernatant was collected and evaporated using SpeedVac (Savant SPD1030, Thermo Scientific, USA). Dried samples were reconstituted in 100 µL of 80% methanol, mixed for 1 min, and centrifuged again at 14, 8000 rpm for 5 min at 4 °C. 80 µL of the new supernatant was collected and stored at − 80 °C until HPLC–MS/MS analysis.

### Reverse phase liquid chromatography-quadrupole-time of flight mass spectrometry (RP/HPLC-qTOF MS/MS) analysis

Aliquots of 2 μL (for both ESI (+) and ESI (−) modes) were injected into a LC–MS/MS system consisting of a quadrupole Q-TOF series 6540 coupled to a HPLC (model 1290) both from Agilent Technologies (Germany), equipped with an Agilent Jet Stream (AJS) thermal orthogonal ESI source. MS control, data acquisition, and data analysis were carried out using the Agilent Mass Hunter Qualitative Analysis software (B.10.0). For the chromatographic separation, an Eclipe Plus C18 analytical column (100 × 2.1 mm, particle size 1.8 μm) with a C18 guard column (0.5 cm × 2.1 mm, particle size 1.8 μm), both from Agilent (Germany) were employed. The column temperature was held at 40 °C and the flow rate was set to 0.5 mL/min. Both ESI (+) and ESI (−) modes used water (LC–MS grade) as mobile phase (A) and ACN as mobile phase (B), and formic acid was used as mobile phase modifier (0.1% for ESI (+) and 0.01% for ESI (−)). The gradient started at 0 min with 0% (B), 0–30% (B) in 7 min, 30–80% (B) in 2 min, 80–100% (B) in 2 min, 100% (B) in 2 min, and 3 min of post-time to come back to initial conditions. The mass spectrometer was operated using the following parameters: capillary voltage of 3000 V (+) or − 3000 V (−), and with a *m/z* range from 25 to 1100. Nebulizer pressure was set at 40 psig and the drying gas flow rate was fixed to 8 L/min and 300 °C. The sheath gas flow was 11 L/min at 350 °C. 110 V was chosen for the fragmentor voltage, whereas the skimmer and octapole voltage were 45 V and 750 V, respectively. MS/MS analyses were performed employing the auto MS/MS mode using 5 precursor per cycle, dynamic exclusion after two spectra (released after 0.5 min), and collision energies of 20 and 40 V. For proper mass accuracy, spectra were corrected using ions *m/z* 121.0509 (C_5_H_4_N_4_) and 922.0098 (C_18_H_18_O_6_N_3_P_3_F_24_) in ESI (+), and *m*/*z* 119.0363 (C_5_H_4_N_4_) and 966.0007 (C_18_H_18_O_6_N_3_P_3_F_24_ + formate) in ESI (−), simultaneously pumped into the ionization source.

### Quality control

Quality control was assured by: i) randomization of the sequence; ii) procedure blank analysis; iii) injection of pool samples to equilibrate the LC–MS system before and after of the different sequence of samples; and iv) injection of standard mixture for checking the retention time, peak shape, intensity and mass accuracy.

### Data processing

LC–MS raw data files were firstly converted to ABF format using Reifycs Abf (Analysis Base File) Converter (accessible at: http://www.reifycs.com/AbfConverter/). Data processing was then performed using MS-DIAL (v. 4.12) software for deconvolution, peak picking, alignment, and identification^43^ using the following parameters: retention time begin, 0 min; retention time end, 14 min; mass range begin, 0 Da; mass range end, 1100 Da; MS1 tolerance, 0.01 Da; smoothing level, 3 scans; minimum peak width, 5 scans; minimum peak height, 1000 amplitude; mass slice width, 0.1 Da; sigma window value for deconvolution, 0.1; accurate mass tolerance for MSP library, 0.01 Da; identification score cut off for MSP library, 80%; retention time tolerance for alignment, 0.1 min; MS1 tolerance for alignment, 0.015 Da. Peak height calculation was performed by combining data for different detected molecular species for each particular compound ([M+H]^+^, [M+NH4]^+^, [M+Na]^+^, [M+K]^+^, [2M+H]^+^, [2M+NH4]^+^, [2M+Na]^+^, [2M+K]^+^ adducts in positive mode, and [M−H]^−^, [2M−H]^−^, [M+Cl]^−^, [M+FA-H]^−^ adducts in negative mode). The MSP file used for annotation was generated by combining MS/MS spectra from NIST20 MS/MS database, the LipidBLAST mass spectral library^44^, and the MassBank of NorthAmerica database (MoNA, available at https://mona.fiehnlab.ucdavis.edu/downloads). All metabolite were annotated following the Metabolomics Standard Initiative (MSI) guidelines^45,46^ as MSI level 2a (metabolites with precursor m/z and MS/MS spectral library matching).

### Data post-processing and statistical analysis

The list of metabolites obtained on each ESI ionization mode was filtered removing unknown metabolites, metabolites with a maximum height below 1000 units or metabolites with a maximum height below three times the average height in the extraction blanks, the Vacutainer® blood collection tube blanks, the Ficoll-Hypaque blanks. Metabolites present in more than 50% of the samples for at least one group were retained. Missing values were imputed by half of the minimum height value, and the data were processed using the bioinformatic tool MS-FLO (https://msflo.fiehnlab.ucdavis.edu/#/)^47^. Duplicated metabolites and isotopes were removed, the height of the different adducts from the same compound was combined, and Systematic Error Removal using Random Forest normalization^48^ using the pool samples as reference samples was applied.

The statistical analysis was performed according to two different experimental designs. In the first one, samples collected from patients at hospital admission were analysed together to search for metabolic biomarkers of prognosis. Multivariate analysis (PCA and PLS-DA) of these samples were performed after “Auto scaling” normalization by using MetaboAnalyst 5.0 web-based software^49^. PLS-DA models were evaluated according to the “Leave-one-out” cross-validation method of R^2^ and Q^2^, and variable importance in projection (VIP) scores were obtained and considered significant when VIP scores > 1.5. Correlation analyses were evaluated by the Pearson’s correlation coefficient (r), heat map representation were obtained, and all groups were compared by using the non-parametric ANOVA (Kruskal Wallis) test. In addition, fold changes between the different COVID-19 positive groups (asymptomatic, mild disease, severe disease, deceased) and the negative control group (non-COVID) were calculated and evaluated by using the non-parametric Mann–Whitney U test. Metabolites were considered significantly altered when raw p-value < 0.05. Patterns of metabolite fold change ratios between the different COVID-19 positive groups and the negative control group (non-COVID) were also investigated using the fuzzy c-means clustering algorithm^50^. For this analysis, different combinations of cluster sizes and fuzzification parameters were explored, and found optimal partitioning with c = 8 and m = 2. These values avoid the appearance of empty clusters and reduced the minimum distance to cluster centroid. Moreover, a threshold for membership values was set to 0.7 for cluster assignation.

The second experimental design consisted on the analysis and comparison of plasma samples from patients belonging to the mild disease group at two different time points (at hospital admission and after 2–3 months of hospital discharge) with the aim of searching for metabolic biomarkers of illness recovery. For this comparison, PCA, PLS-DA, and the paired non-parametric Mann–Whitney U test were applied.

### Data visualization, enrichment and pathway analysis

Data matrices obtained after the comparison of COVID-19 positive samples against the negative controls were combined to generate a joint dataset. For those metabolites detected in both ESI (+) and ESI (−) modes, data with the highest similarity score (from the MSP file), highest peak intensity, and/or better peak shape were retained.

For metabolic network mapping, the InChiKey or compound names were imported into the web-based Chemical Translation Service (http://cts.fiehnlab.ucdavis.edu/batch)^51^ to obtain the PubChem Compound Identifiers (CID) and the Kyoto Encyclopedia of Genes and Genomes identifiers (KEGG ID). Simplified molecular-input line-entry system (SMILES) codes were obtained from the MSP file or from the PubChem Compound Identifier Exchange service (https://pubchem.ncbi.nlm.nih.gov/idexchange/idexchange.cgi), and chemical similarity enrichment calculations were done using ChemRICH^52^. KEGG reactant pairs and Tanimoto similarity calculations (using a threshold of 0.7) were done using MetaMapp^53^. The final network graph was imported into Cytoscape 3.7.2^54^, as well as the results generated in MetaboAnalyst. The graphs were visualized using a yED organic layout algorithm in Cytoscape. Metabolite enrichment analysis was performed using the MBROLE 2.0. web-based software^55^. Significantly altered metabolites matching the KEGG database were imported and overrepresentation analysis against the KEGG pathway module, and using *Homo sapiens* as the background set, was performed. Annotations with p-values lower than 0.05 were considered significantly enriched.

## Supplementary Information

Supplementary Figures.

Supplementary Tables.
