# Supplementary material for: Metabolomics study of COVID-19 patients in four different clinical stages
Source: Sci Rep. 2022 Jan 31;12:1650. doi: 10.1038/s41598-022-05667-0 (PMC8803913; doi:10.1038/s41598-022-05667-0)

Supplementary Information for

Metabolomics study of COVID-19 patients in four different clinical stages

Alberto Valdés[1], Lorena Ortega Moreno[2,3,4], Silvia Rojo Rello[5], Antonio Orduña[5,6], David Bernardo[4,7], Alejandro Cifuentes[1]*

[1]Laboratory of Foodomics, Institute of Food Science Research, CIAL, CSIC, Nicolás Cabrera 9, Madrid, 28049, Spain

[2]Universidad Autónoma de Madrid. Dpt. Medicina. Madrid.

[3]Instituto de Investigación Sanitaria Hospital Universitario de La Princesa. Madrid.

[4]Centro de Investigación Biomédica en Red (CIBERehd).

[5]Servicio de Microbiología, Hospital Clínico Universitario de Valladolid, Valladolid, 47004, Spain.

[6]Departmento de Microbiología. Universidad de Valladolid. Valladolid, Spain.

[7]Unidad de Excelencia Instituto de Biomedicina y Genética Molecular (IBGM), Universidad de Valladolid-CSIC. Valladolid. Spain.

* Corresponding author:

a.cifuentes@csic.es (AC); ORCID: 0000-0002-7464-0217

Table of contents

Supplementary Figure S1. PCA score plots of data obtained by RP/HPLC-qTOF MS/MS ESI (+) (**A**) and RP/HPLC-qTOF MS/MS ESI (-) (**B**) from plasma of patients collected at hospital admission.

Supplementary Figure S2. Box-plot representation of metabolites with VIP scores > 1.5 in plasma of COVID-19 positive patients (asymptomatic, mild disease, severe disease and deceased) and non-COVID-19 control patients collected at hospital admission.

Supplementary Figure S3. Heat map representation and sample classification of data obtained by RP/HPLC-qTOF MS/MS ESI (+) (**A**) and RP/HPLC-qTOF MS/MS ESI (-) (**B**) from plasma of patients collected at hospital admission.

Supplementary Figure S4. MetaMapp visualization of metabolomics data highlighting the differential metabolic regulation in asymptomatic COVID-19 positive patients compared to the non-COVID control group. Red edges denote KEGG reactant pair links and light blue edges symbolize Tanimoto chemical similarity at T > 700. Node sizes reflect fold change. Metabolites found significantly increased are given as red nodes, and blue nodes denotes decreased metabolites (significance determined using Mann-Whitney U test with p-value < 0.05). Metabolites not significantly altered are given as yellow nodes.

Supplementary Figure S5. MetaMapp visualization of metabolomics data highlighting the differential metabolic regulation in mild disease COVID-19 positive patients compared to the non-COVID control group. Red edges denote KEGG reactant pair links and light blue edges symbolize Tanimoto chemical similarity at T > 700. Node sizes reflect fold change. Metabolites found significantly increased are given as red nodes, and blue nodes denotes decreased metabolites (significance determined using Mann-Whitney U test with p-value < 0.05). Metabolites not significantly altered are given as yellow nodes.

Supplementary Figure S6. MetaMapp visualization of metabolomics data highlighting the differential metabolic regulation in severe disease COVID-19 positive patients compared to the non-COVID control group. Red edges denote KEGG reactant pair links and light blue edges symbolize Tanimoto chemical similarity at T > 700. Node sizes reflect fold change. Metabolites found significantly increased are given as red nodes, and blue nodes denotes decreased metabolites (significance determined using Mann-Whitney U test with p-value < 0.05). Metabolites not significantly altered are given as yellow nodes.

Supplementary Figure S7. MetaMapp visualization of metabolomics data highlighting the differential metabolic regulation in deceased COVID-19 positive patients compared to the non-COVID control group. Red edges denote KEGG reactant pair links and light blue edges symbolize Tanimoto chemical similarity at T > 700. Node sizes reflect fold change. Metabolites found significantly increased are given as red nodes, and blue nodes denotes decreased metabolites (significance determined using Mann-Whitney U test with p-value < 0.05). Metabolites not significantly altered are given as yellow nodes.

Supplementary Figure S8. PCA score plots of data obtained by RP/HPLC-qTOF MS/MS ESI (+) (**A**) and RP/HPLC-qTOF MS/MS ESI (-) (**B**) from plasma of mild disease COVID-19 positive patients at hospital admission (0) and the same patients after 2-3 months of hospital discharge (1).

Supplementary Figure S9. PLS-DA score plots of data obtained by RP/HPLC-qTOF MS/MS ESI (+) (**A**) and RP/HPLC-qTOF MS/MS ESI (-) (**B**) from plasma of mild disease COVID-19 positive patients at hospital admission (0) and the same patients after 2-3 months of hospital discharge (1).

Supplementary Figure S10. Box-plot representation of metabolites with VIP scores > 1.5 in the comparison of plasma of mild disease COVID-19 positive patients at hospital admission and after 2-3 months of hospital discharge. non-COVID-19 control patients were include for graphical comparison.

Supplementary Table S1. Lists of annotated metabolites in RP/HPLC-qTOF MS/MS ESI (+) data from plasma of patients collected at hospital admission, including the statistical values for the different analyses (ANOVA, U test and PLS-DA).

Supplementary Table S2. Lists of annotated metabolites in RP/HPLC-qTOF MS/MS ESI (-) data from plasma of patients collected at hospital admission, including the statistical values for the different analyses (ANOVA, U test and PLS-DA).

Supplementary Table S3. Pearson correlation (r) values for annotated metabolites in plasma of patients collected at hospital admission and obtained by RP/HPLC-qTOF MS/MS ESI (+).

Supplementary Table S4. Pearson correlation p-values for annotated metabolites in plasma of patients collected at hospital admission and obtained by RP/HPLC-qTOF MS/MS ESI (+).

Supplementary Table S5. Pearson correlation (r) values for annotated metabolites in plasma of patients collected at hospital admission and obtained by RP/HPLC-qTOF MS/MS ESI (-).

Supplementary Table S6. Pearson correlation p-values for annotated metabolites in plasma of patients collected at hospital admission and obtained by RP/HPLC-qTOF MS/MS ESI (-).

Supplementary Table S7. MFuzz membership values of annotated metabolites in plasma of patients collected at hospital admission.

Supplementary Table S8. MFuzz cluster composition (with a membership value > 0.7) of annotated metabolites in plasma of patients collected at hospital admission.

Supplementary Table S9. ChemRICH results obtained after comparing mild disease COVID-19 positive patients and the non-COVID control group.

Supplementary Table S10. ChemRICH results obtained after comparing severe disease COVID-19 positive patients and the non-COVID control group.

Supplementary Table S11. ChemRICH results obtained after comparing deceased COVID-19 positive patients and the non-COVID control group.

Supplementary Table S12. Statistical values of the paired U test and PLS-DA for annotated metabolites in RP/HPLC-qTOF MS/MS ESI (+) after comparing the mild disease COVID-19 positive patients at hospital admission and after 2-3 months of hospital discharge.

Supplementary Table S13. Statistical values of the paired U test and PLS-DA for annotated metabolites in RP/HPLC-qTOF MS/MS ESI (-) after comparing the mild disease COVID-19 positive patients at hospital admission and after 2-3 months of hospital discharge.

Supplementary Table S14. ChemRICH results obtained after comparing mild disease COVID-19 positive patients at hospital admission and after 2-3 months of hospital discharge.

Supplementary Table S15. Significantly enriched KEGG human metabolic pathways (in dark shade) from the analysis of significantly altered metabolites (after Mann-Whitney U test with p-value < 0.05) in mild disease COVID-19 positive patients at hospital admission and after 2-3 months of hospital discharge.

Supplementary Table S16. Baseline patient’s characteristics (demographics, co-morbidities and treatments) of the study.

Supplementary Figure S1.

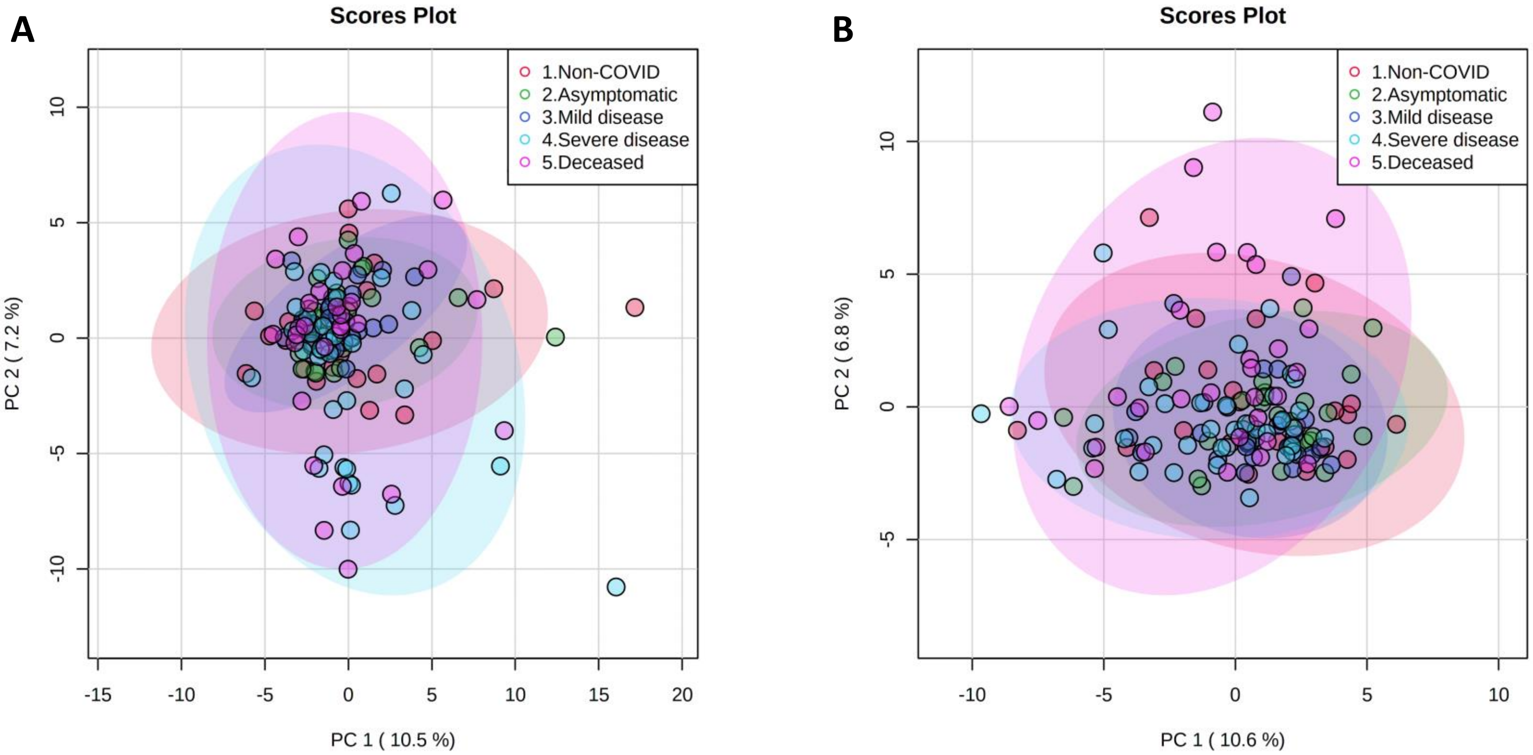

Supplementary Figure S2.

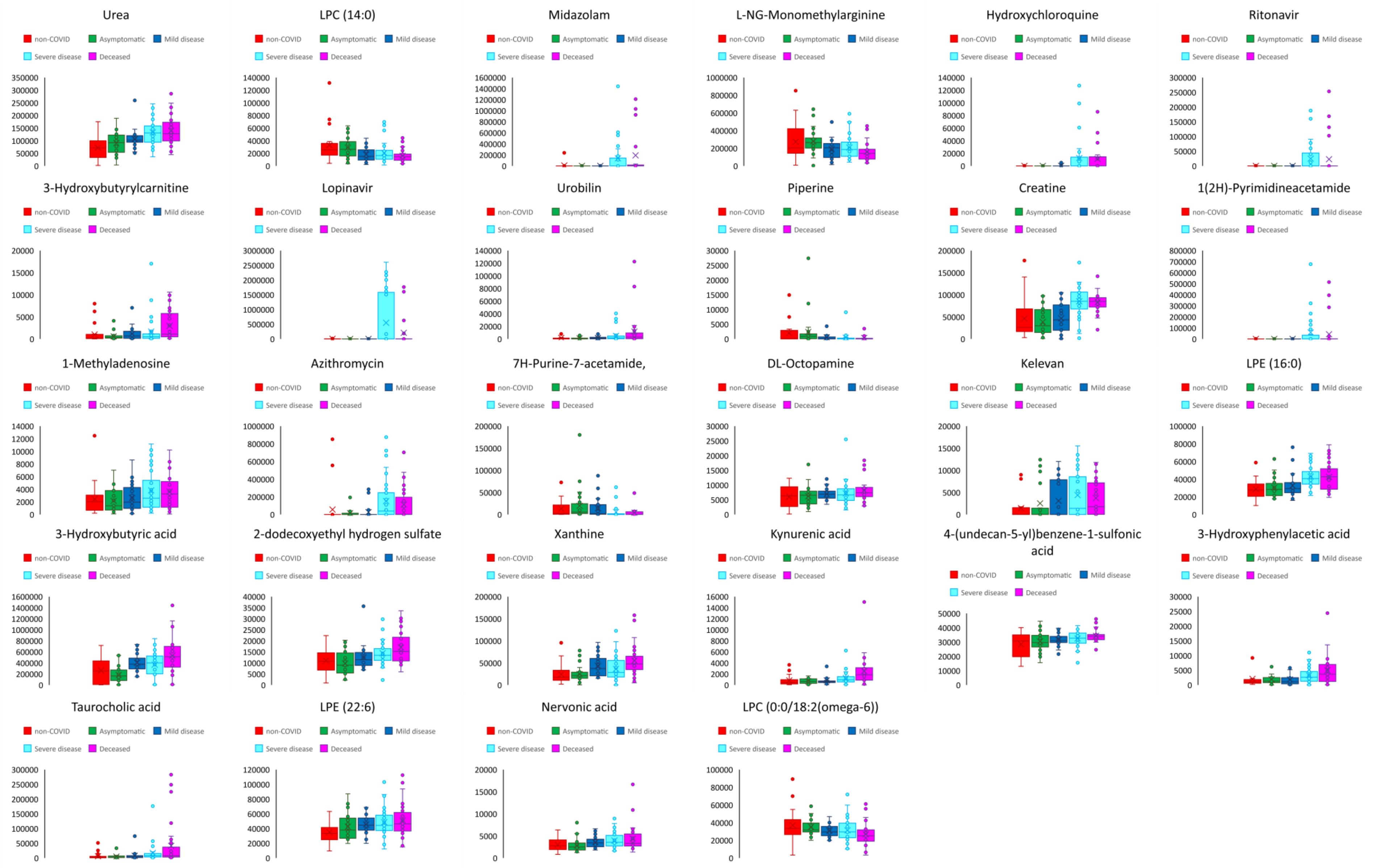

Supplementary Figure S3.

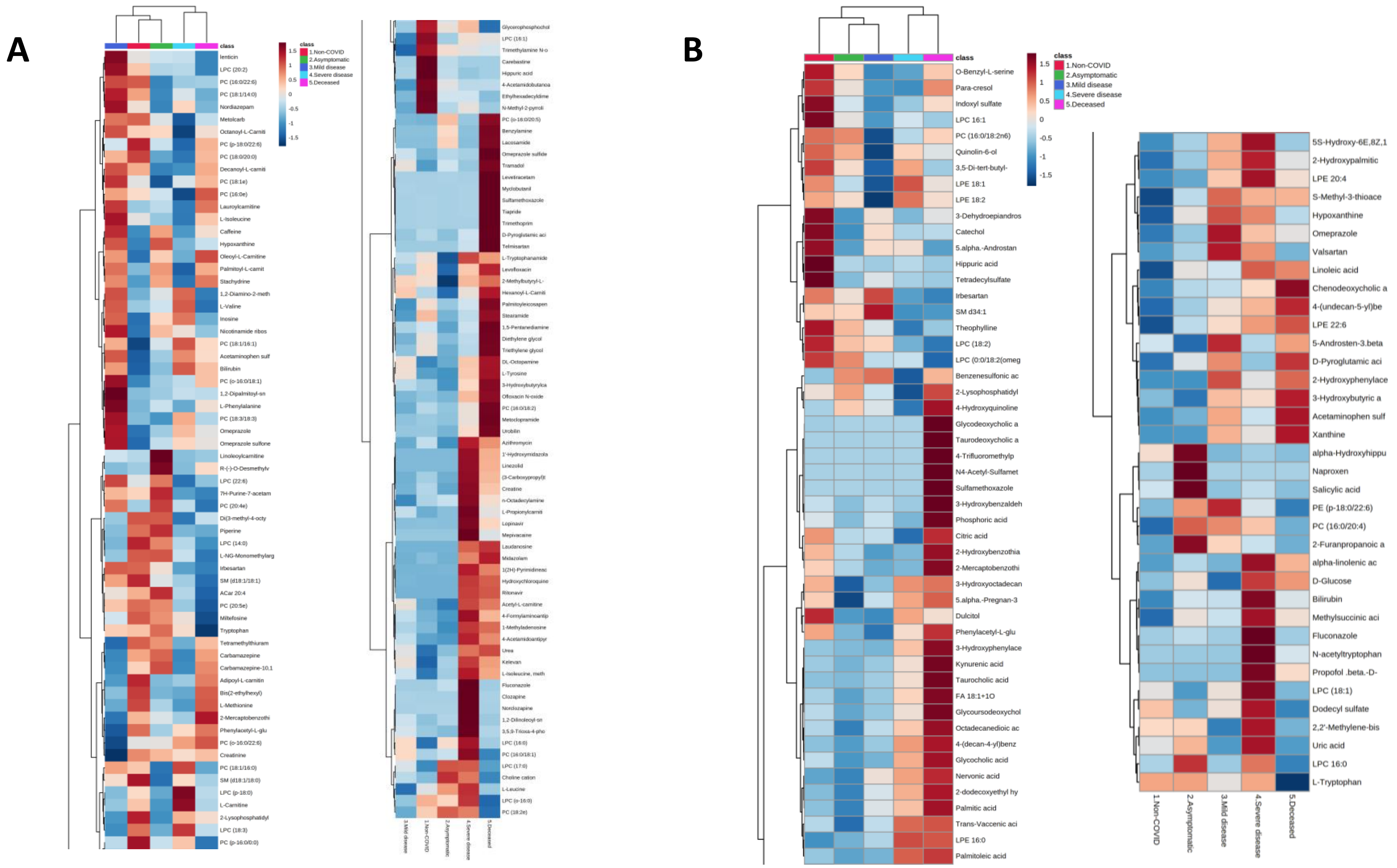

Supplementary Figure S4.

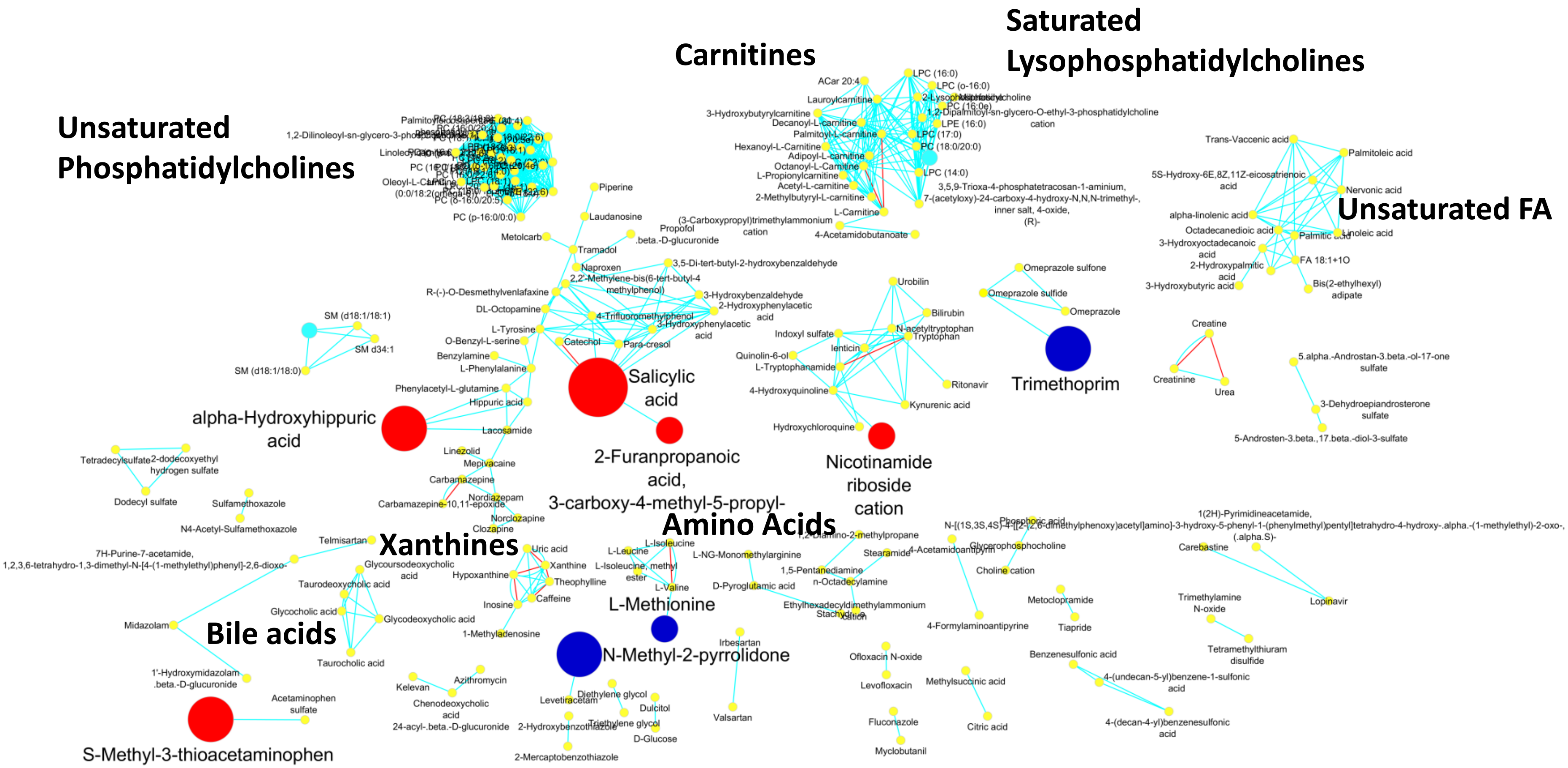

Supplementary Figure S5.

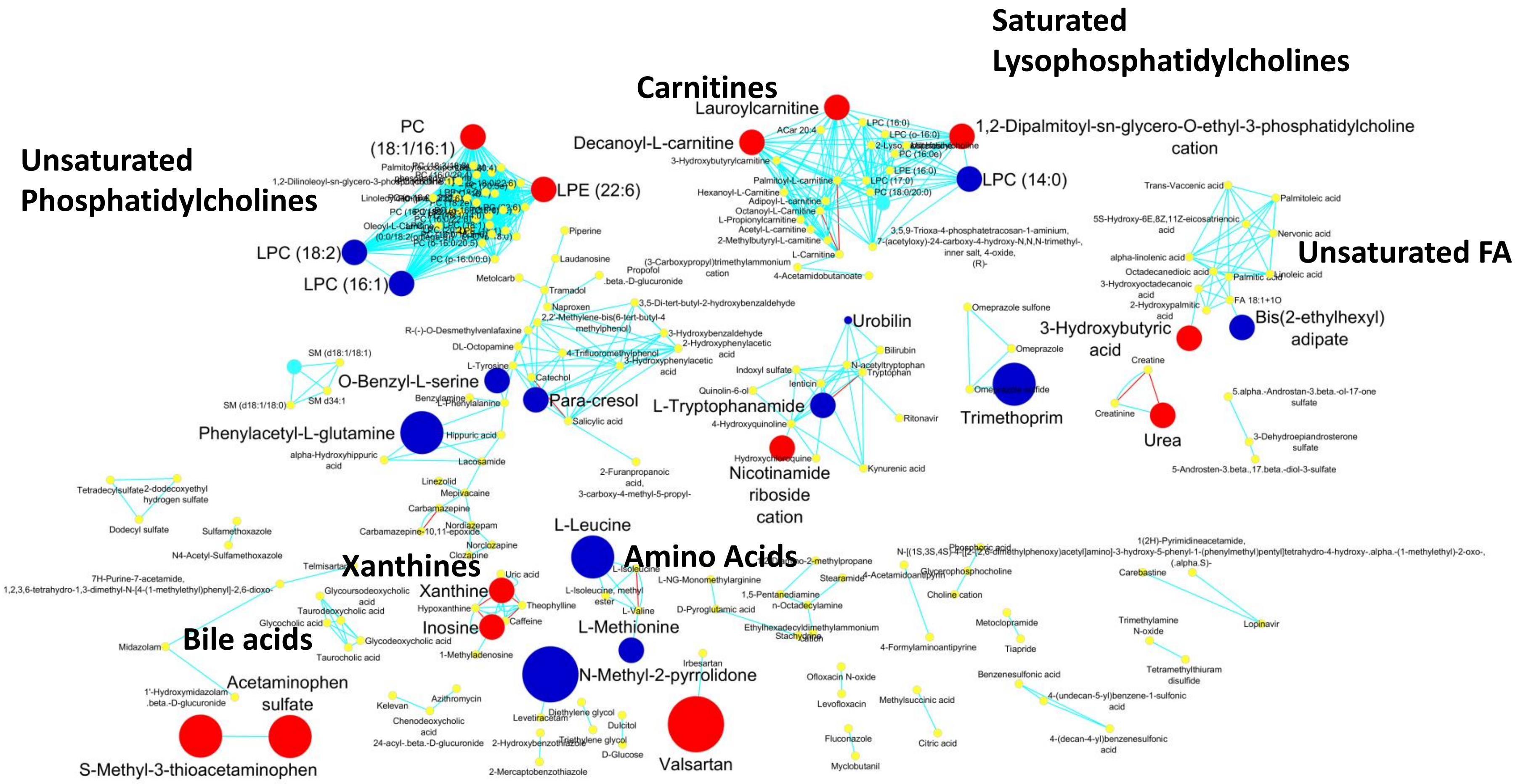

Supplementary Figure S6.

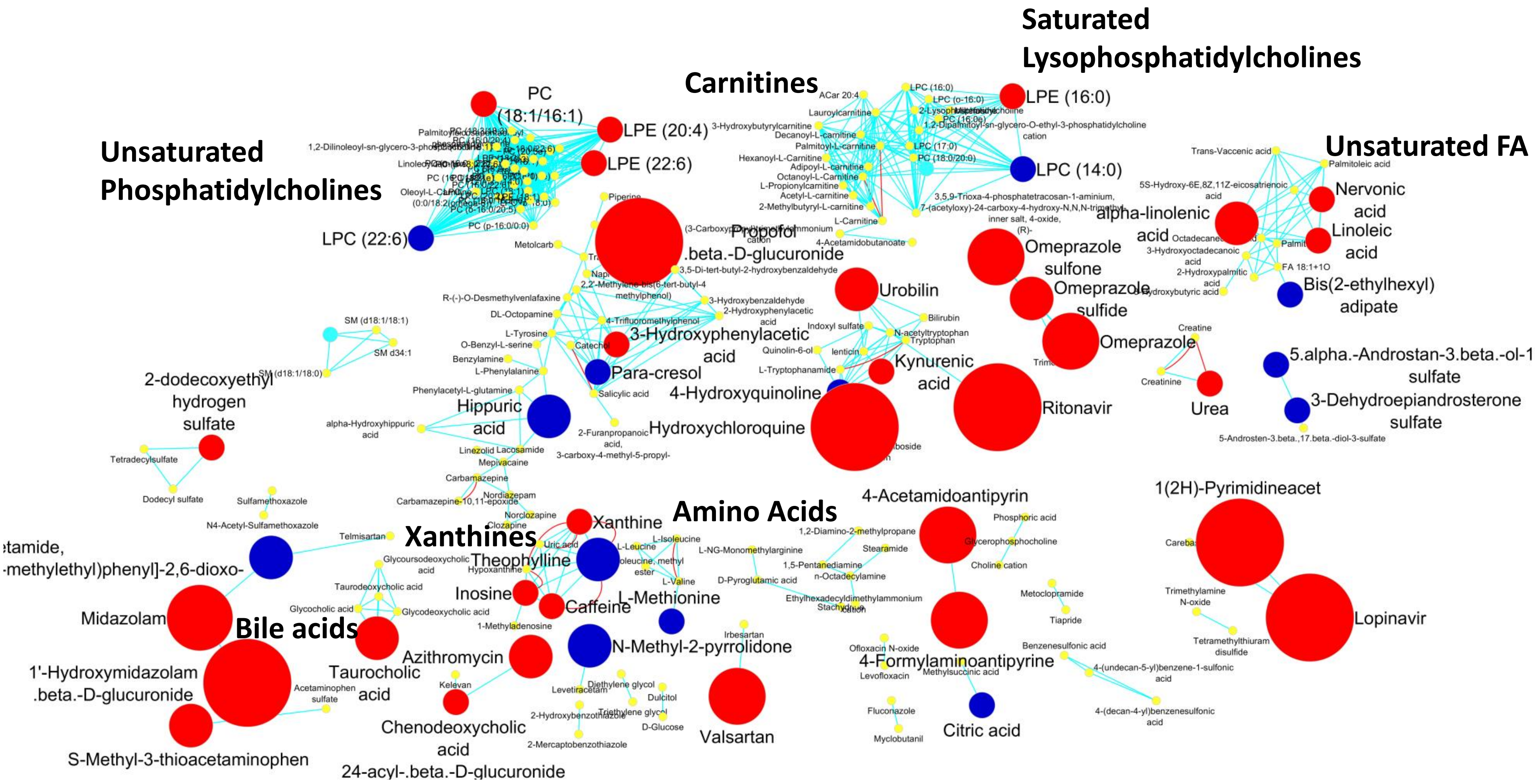

Supplementary Figure S7.

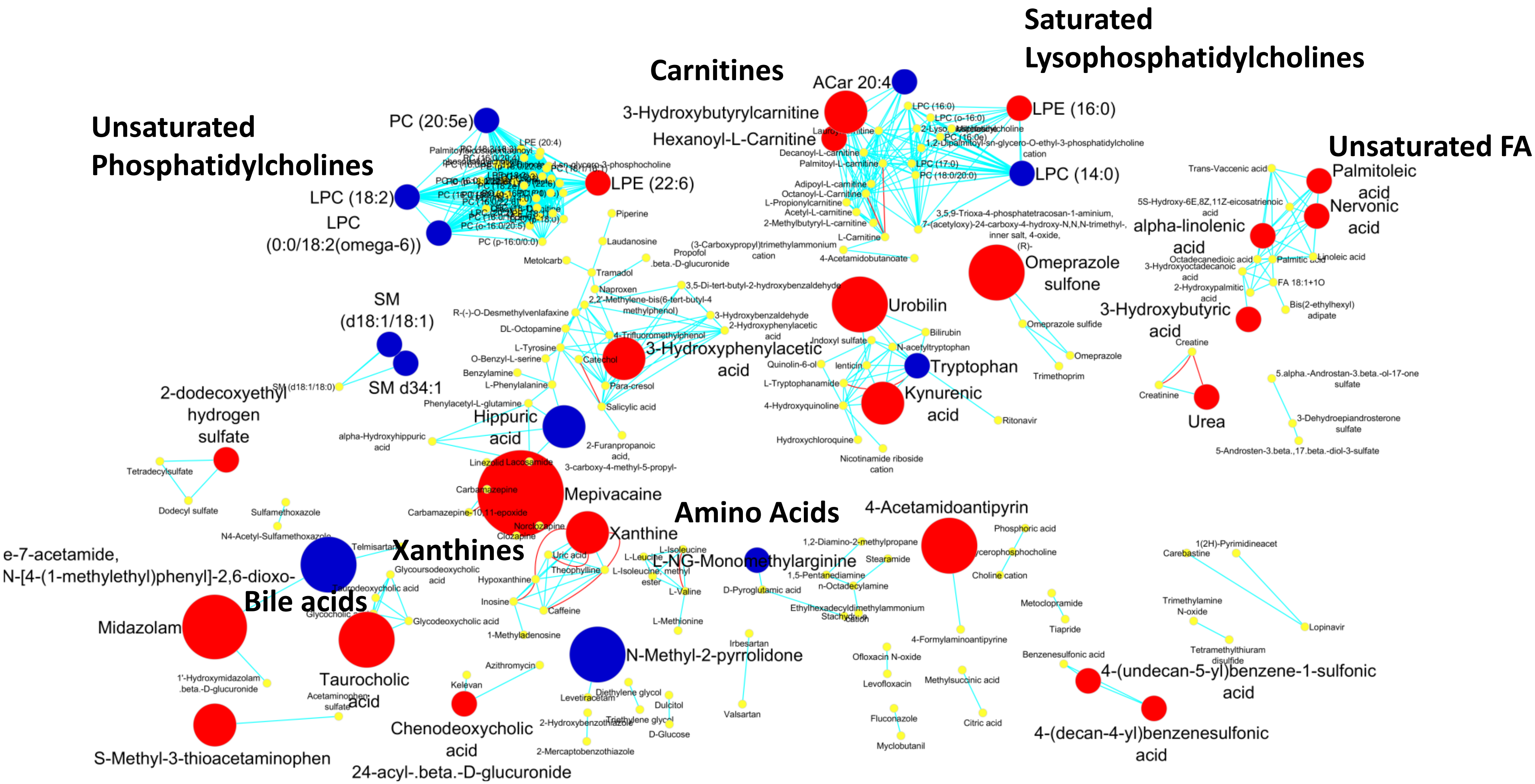

Supplementary Figure S8.

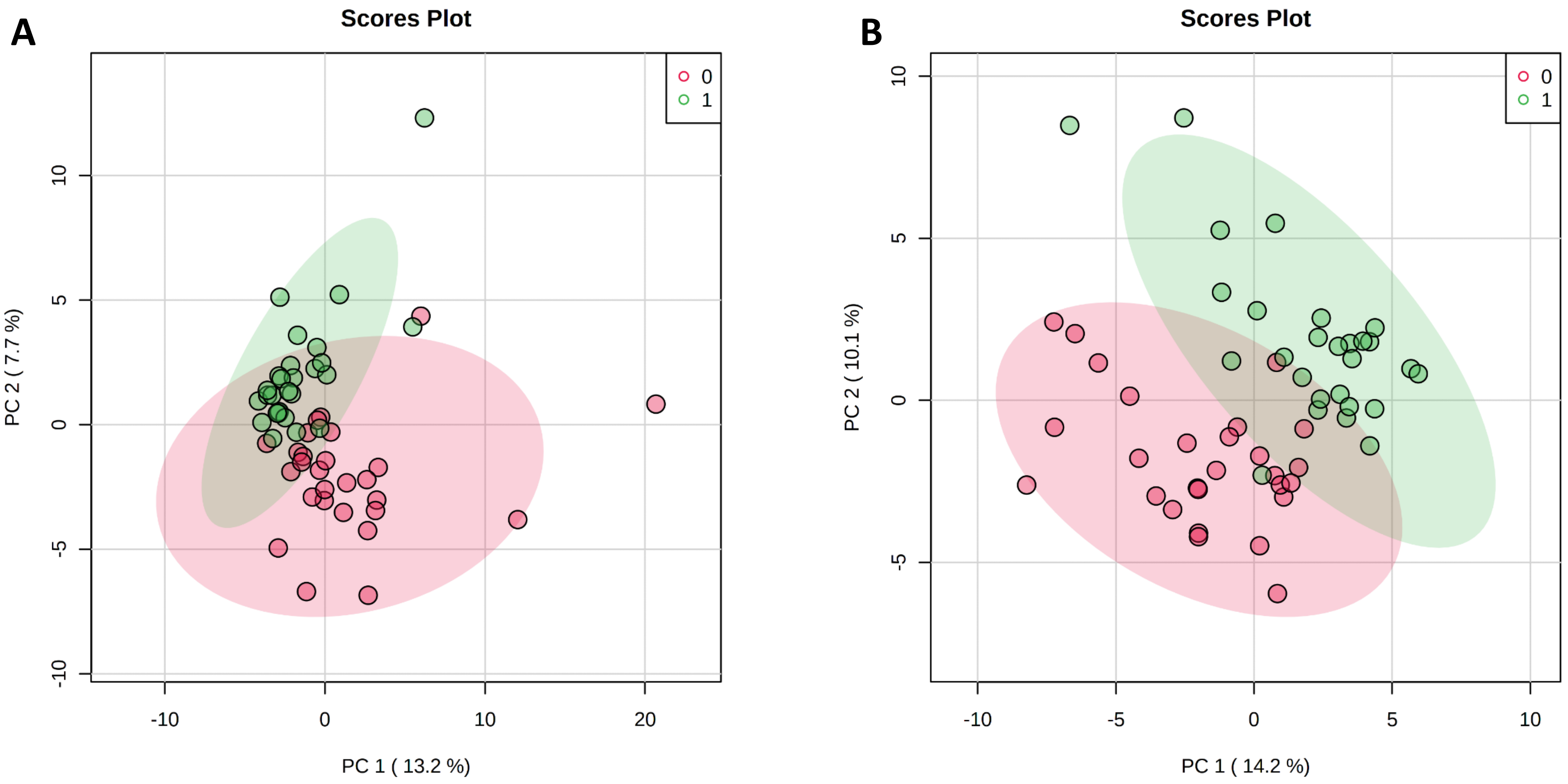

Supplementary Figure S9.

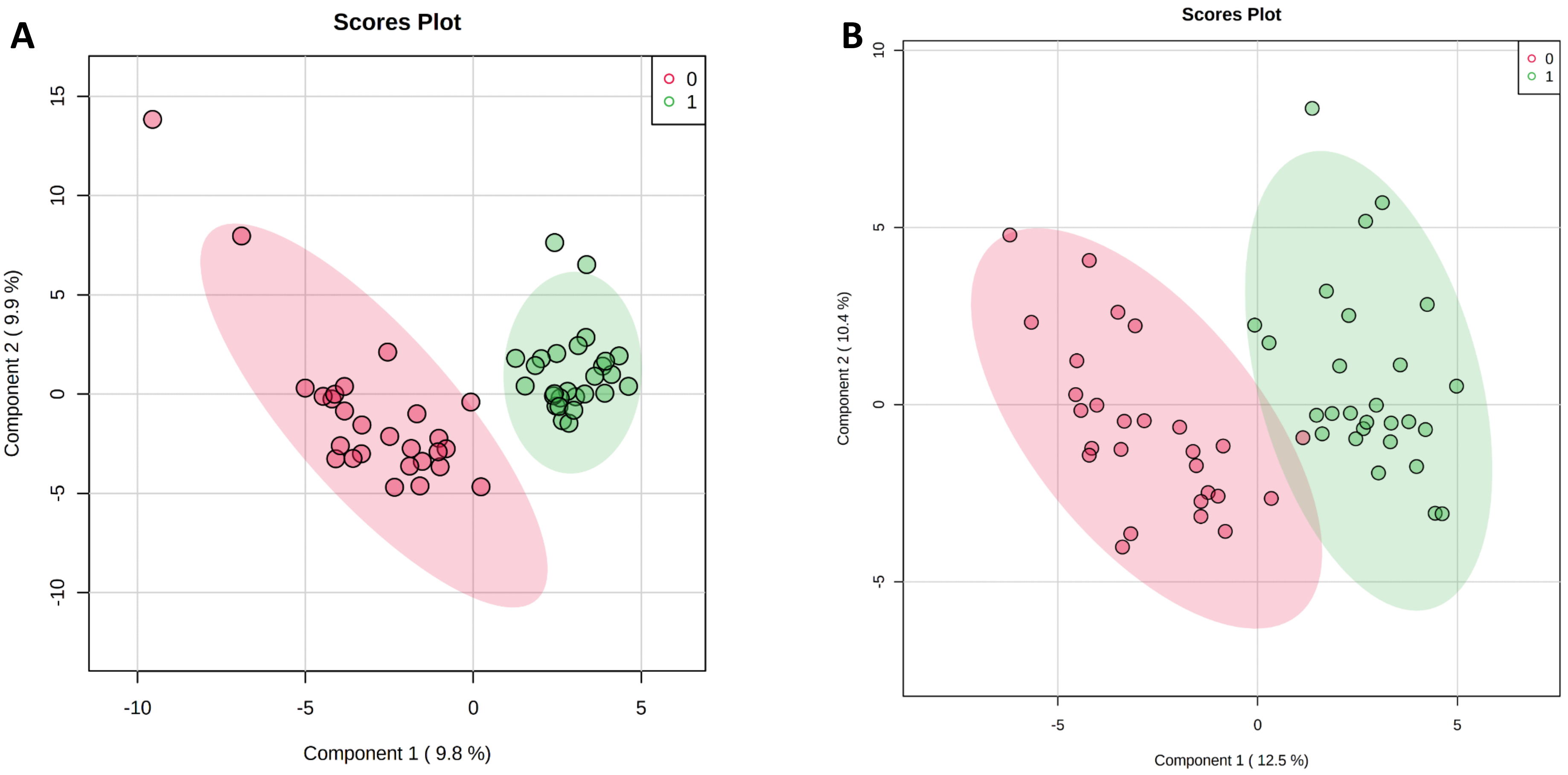

Supplementary Figure S10.

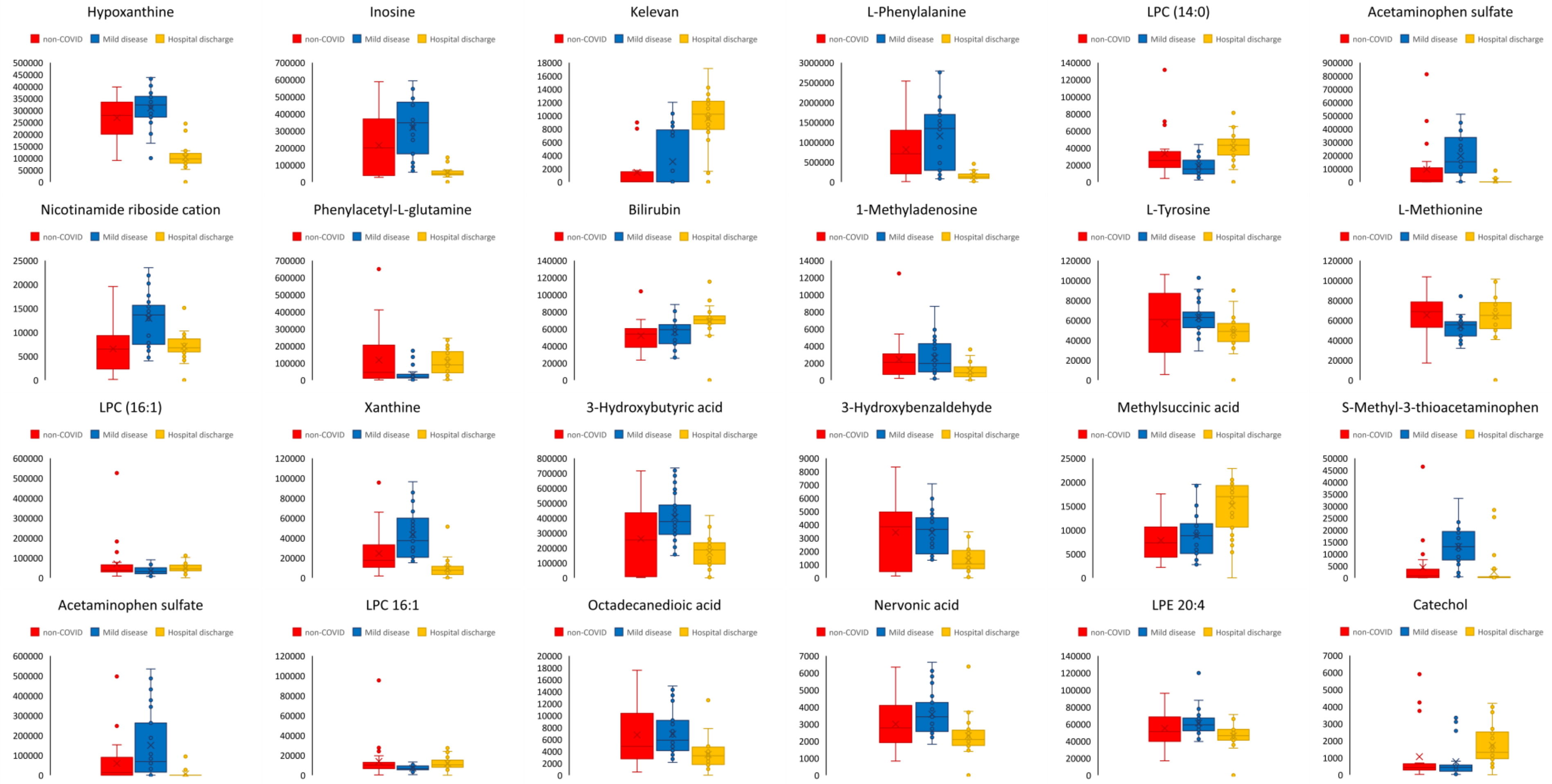

Supplement: Supplementary file 1 — Supplementary Figures. [file 41598_2022_5667_MOESM1_ESM.pdf]
